# Cryoneurolysis of the Subcostal Nerve: A Technical Description and Case Report

**DOI:** 10.7759/cureus.57521

**Published:** 2024-04-03

**Authors:** Arun Kalava, Karen Pham, Sidney Okon

**Affiliations:** 1 Anesthesiology, University of Central Florida, Orlando, USA; 2 Anesthesiology, TampaPainMD, Tampa, USA; 3 Anesthesiology, Northeast Ohio Medical University, Rootstown, USA; 4 College of Arts and Sciences, University of Pennsylvania, Philadelphia, USA; 5 Medicine, Mercy Catholic Medical Center, Philadelphia, USA

**Keywords:** peripheral neuropathy, regional anesthesia and chronic pain, ultrasound-guided, sensory block, lower thoracic back pain, chronic and acute pain management, cryoanalgesia, pain, subcostal nerve, cryoneurolysis

## Abstract

Cryoneurolysis has been utilized for numerous persistent and intractable painful conditions, including phantom limb pain and postsurgical pain. Although there are reports on the effectiveness of cryoneurolysis in various regions, including the intercostal nerves, the subcostal nerve remains a common culprit of chronic pain for which the literature is scarce. Different modalities are commonly utilized to address subcostal neuropathic pain, such as non-opioid pharmacotherapy, including nonsteroidal anti-inflammatory drugs (NSAIDs) and anticonvulsants, site-specific regional anesthesia, and radiofrequency ablation.However, the analgesia provided by these modalities is often inadequate or short-lived. Cryoneurolysis of the subcostal nerve remains largely unexplored and may provide a promising solution.Here, we present the first technical description of ultrasound and fluoroscopic guided percutaneous cryoneurolysis of the subcostal nerve and the case of a patient with 14 years of lower thoracic rib pain who failed multiple interventions but achieved complete pain resolution at the three-month follow-up through this procedure.

## Introduction

Cryoneurolysis has been utilized for numerous persistent and intractable painful conditions, including phantom limb pain and postsurgical pain following thoracotomy, mastectomy, and skin graft harvesting [1-3]. There are extensive reports on the effectiveness of cryoneurolysis in various regions, including the intercostal nerves [4-7]. However, the subcostal nerve remains a common area of chronic pain for which the literature is scarce. 

While cryoneurolysis is referenced as a more modern form of treatment, the practice of cryoanalgesia dates to the era of Hippocrates (460-377 BC) when snow was applied to wounds to relieve pain and inflammation [8,9]. Modern-day cryoanalgesia relies on advancements in technology that have allowed us to achieve temperatures as cold as -196 °C in the reversible ablation of nerves [5]. The rapid expansion of nitrous oxide or carbon dioxide within a cryoneurolysis probe produces an ice ball at the tip of the cannula that encompasses the target nerve. Between −20 and −100 °C, Wallerian degeneration occurs, which leads to the cessation of nerve conduction and the sensation of pain [3,5]. 

In 1976, Lloyd et al. demonstrated the superiority of cryoneurolysis to neuroablation with phenol, alcohol, and surgical lesions in the treatment of intercostal, low back, facial, and neoplastic pain [10]. Peripheral mononeuropathies resistant to standard treatment have become increasingly treated with cryoneurolysis. These include mononeuropathies of the intercostals, genicular, iliohypogastric, ilioinguinal, pudendal, lateral femoral cutaneous, and sacral nerve roots [11]. Despite the pervasiveness of subcostal neuralgia, there are scarce reports of the possible benefits of cryoneurolysis of this specific region [5].

The subcostal nerve, the largest among the thoracic spinal nerves in diameter, travels below the 12th rib, passes behind the kidney, and gives rise to branches, including the lateral cutaneous nerve and the motor branch to pyramidalis [12]. The subcostal nerve, unlike the intercostal nerves, is especially susceptible to injury as it passes beyond the tips of intact ribs that could otherwise shield the nerve from damage. Injury or compression of the subcostal nerve is commonly due to repeated squeezing of the nerve between the tips of the ribs and the iliac crest [13]. However, there have also been reports of iatrogenic injury of the subcostal nerve, such as during laparoscopic surgery for abdominal lipoma excision [14]. 

In cryoneurolysis of the subcostal nerve, a cryo-needle is passed through the skin, subcutaneous fat, and muscle to reach the subcostal nerve.

## Case presentation

**Figure 1:**
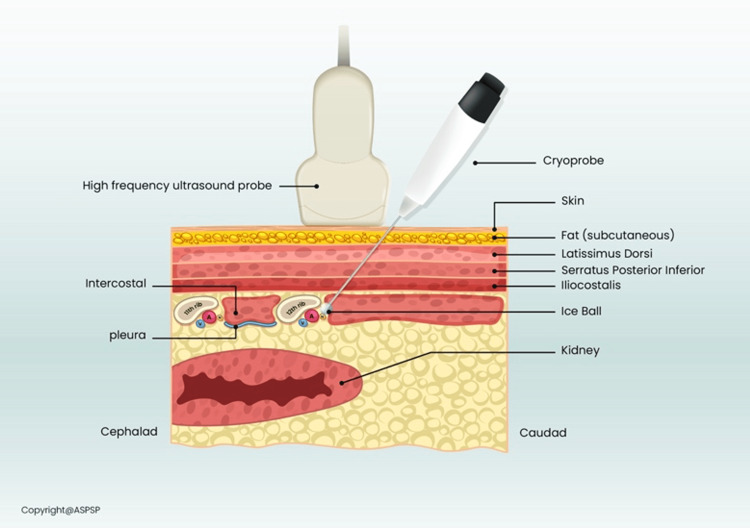
An illustration of the cryoneurolysis needle and the structures it passes through. Permission granted by the American Society for Post Surgical Pain (ASPSP).

A 69-year-old male with a past medical history of glaucoma, hypertension, coronary artery bypass graft, and lumbar/thoracic laminectomy presented with chronic left posterior lower thoracic rib pain persisting for 14 years, without any history of prior trauma. The patient described his pain as an 8 out of 10, unrelenting, dull ache in his lower back. The pain was present at rest and exacerbated by inspiration, movement, and applied pressure. The pain persisted through the night and prevented him from sleeping on his left side. On physical exam, there was point tenderness to palpation in the vicinity of the 12th rib on the lateral posterior thoracic wall. Previous therapeutic interventions attempted by the patient included nonsteroidal anti-inflammatory drugs (NSAIDs), ice, heat, and thoracic epidural steroid injections performed by other pain physicians. Notably, ice and heat provided moderate relief, while the other interventions had minimal efficacy (Figure 1).

**Figure 2:**
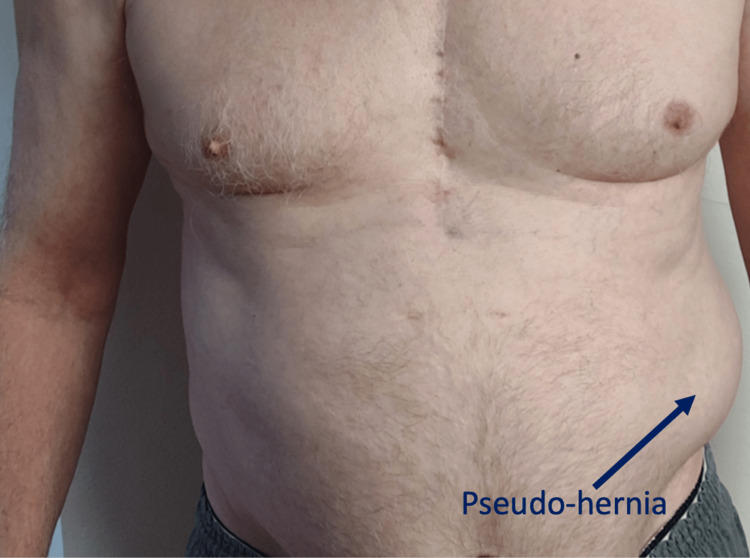
An abdominal pseudo-hernia observed in the patient.

The patient received three ultrasound-guided subcostal nerve blocks that were both diagnostic and therapeutic. Each nerve block provided 100% pain relief for two to three weeks. The nerve blocks were well tolerated and without complications, except for a self-limiting pseudo-hernia that manifested following the first of the three blocks (Figure 2). This subcostal bulge is a known consequence of loss of tone from the external oblique muscle, which resolves with the return of sensation [15]. With these results, the patient was agreeable to cryoneurolysis of the subcostal nerve, given the documented ability of cryoneurolysis to provide up to one year of relief in the treatment of similar peripheral mononeuropathies [6,16].

**Figure 3:**
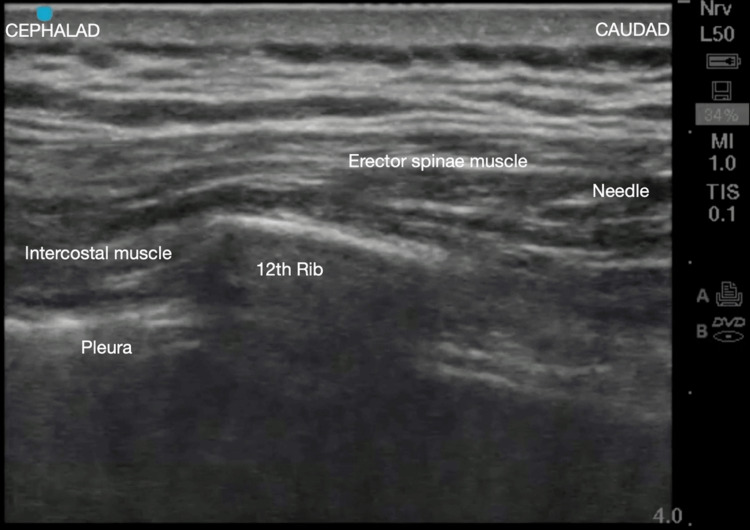
An ultrasound image of the cryoanalgesia needle in the vicinity of the 12th rib and the pleura.

**Figure 4:**
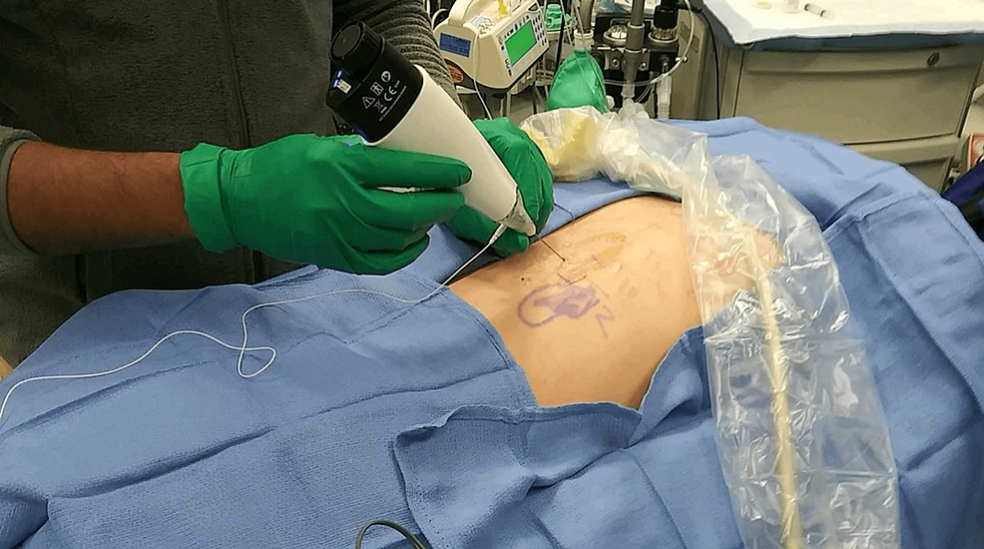
Patient in the prone position and prepared in a sterile fashion. A cryoneurolysis handpiece (Iovera Focused Cold Therapy; Myoscience, Fremont, CA) is being used to deliver -88 ℃ nitrous oxide.

**Figure 5:**
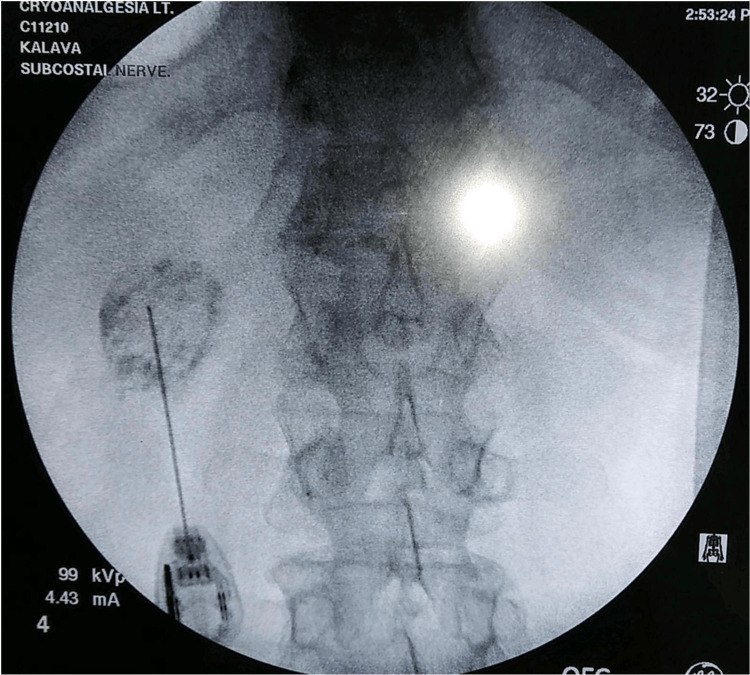
Fluoroscopy-guided imaging of the subcostal nerve cryoneurolysis.

Written informed consent for cryoneurolysis of the subcostal nerve was obtained. The patient was brought into the operating room and sedated with monitored anesthesia care. Positioning of the patient was prone with the upper abdomen supported on pillows and arms placed over the edge of the bed to aid in the lateral retraction of the scapula to expose the rib angles. The ribs were palpated 6 cm lateral to the midline. After the area was prepared with chlorhexidine gluconate and draped in a sterile fashion, the subcostal nerve was localized with a portable ultrasound (SonoSite M-Turbo, Bothell, WA) with a 13-6 MHz linear ultrasound transducer. The ultrasound probe, placed within a sterile sleeve, was positioned in the sagittal plane approximately 4 cm lateral to the spinous process. After the 12th rib and pleura were visualized, a skin wheal was raised with 2 mL of 1% lidocaine. A 20-gauge angiocatheter placed caudal to the transducer was inserted through the skin wheal at approximately 20° cephalad. The needle was advanced until the tip was below the inferior border of the 12th rib, after which 5 mL of 2% lidocaine was deposited to anesthetize the subcostal nerve (Figure 3). A 5.5-cm 22-gauge cryoprobe (Smart Tip 2190, Myoscience, Freemont, CA) was advanced through the angiocatheter (Figure 4). The probe's final paraneural position was confirmed through electrical neurostimulation and fluoroscopic image guidance (Figure 5).

A positive thoracoabdominal twitch was elicited at 1.5 mA. A cryoneurolysis handpiece (Iovera Focused Cold Therapy; Myoscience) was used to deliver -88 ℃ nitrous oxide over 106 seconds. The ramp-up and ramp-down times were swift, including one second of pre-warming time, 60 seconds within the cooling cycle, and 45 seconds of post-cooling. For a more reliable uptake of cryotherapy, a second lesion was performed. The patient tolerated the procedure well, and neither discomfort nor complications were reported after treatment. At the three-week follow-up, the patient reported sustained 100% pain relief, which persisted at three months.

## Discussion

The case discussed here suggests the viability of cryoneurolysis in the treatment of chronic refractory subcostal neuritis with a sustained duration of effect. After multiple failed interventions, including invasive and non-invasive measures, cryoneurolysis was proposed to the patient given its safety profile, longstanding history as an effective, albeit underutilized, form of analgesia, as well as potential for prolonged duration of relief [17]. Moreover, recent studies attest to the clinical significance of cryoneurolysis in the treatment of refractory neuropathic pain [16,18]. 

In 2013, Yoon et al. conducted a prospective study on the efficacy of cryoneurolysis in patients with chronic pain associated with mononeuropathies of the ilioinguinal, posterior tibial, saphenous, gluteal, sural, and geniculate nerves. Twenty-two patients, with a mean age of 49.5 years and a mean pain score of 8.3 ± 1.9 pre-intervention, demonstrated statistically significant changes in pain scores at one month (2.3 ± 2.5), three months (3.2 ± 2.5), six months (4.7 ± 2.7), and 12 months (5.1 ± 3.7) without encountering relevant complications [16].

More recently, Nemecek et al. demonstrated pain reduction of at least 30% in over half of their cohort one month post-cryoneurolysis. The statistical power of their retrospective study at one-month follow-up was 0.99 (effect size of *r* = 0.65; *α* = 0.05; *n* = 24). Notably, these results included data from two patients who received cryoneurolysis of the subcostal nerve [5]. 

Our patient reported 100% pain relief three weeks following cryoneurolysis. His pain score of 0 remained the same at three months. The variable response between our patient and the above-described cohorts is likely due to the subjectivity of self-reported scores, as well as the variable regeneration rate of treated axons. Moreover, the study reported by Nemecek et al. utilized different materials and techniques. Needle probes were not handheld as in our case. The researchers also produced two to three lesions at a temperature of -78 °C per nerve for 2 minutes while we produced lesions at -88 °C over 106 seconds.

Lasting pain relief from cryoneurolysis relies on endoneural edema, creating a conduction block akin to that of local anesthetics. Ice crystals formed at the tip of the cryoprobe lead to extensive vascular damage to the vasa nervorum [15]. Intact endoneurium provides the basis of axon regeneration along the surviving skeleton at a rate of 1-3 mm per day. The duration of the effect of cryoneurolysis is believed to be proportional to the distance between the cryoneurolysis site and terminal branches of the lesioned nerve [5,16]. Ideal treatment parameters for cryoneurolysis, including freeze and thaw duration, number of cycles, and timing of repeat procedures, remain unknown. Further research may illuminate the means to achieve consistent and prolonged treatment effects.

In addition to patients with chronic refractory neuropathic pain, patients with severe postsurgical pain necessitating opioid use may benefit significantly from cryoneurolysis. In a systematic review and meta-analysis of 24 randomized controlled trials, Park et al. found that the adjunct use of cryoanalgesia with opioid medications proved more effective than using opioids alone in treating persistent pain after thoracotomy. Cohorts who received cryoneurolysis also displayed a significant reduction in future reliance on opioids for pain management [19]. 

Contraindications to cryoneurolysis include concurrent use of anticoagulation, localized infection, and disorders relating to cold hypersensitivity, such as Raynaud’s syndrome and cryoglobulinemia. Relative contraindications include a bleeding diathesis site or site such as the face where risks of depigmentation, hyperpigmentation, and alopecia may be of concern [15]. One of the most limiting factors of using cryoneurolysis is the potential for prolonged motor blockade, which may limit a patient’s ADLs; however, the risk is minimal compared to similar invasive measures such as radiofrequency ablation [20]. 

Cryoneurolysis has the potential to achieve astounding results of up to months of analgesia [20]. In contrast to continuous peripheral nerve blocks - the current gold standard of care for prolonged postoperative analgesia - cryoneurolysis eliminates the risk of catheter dislodgement and provides a longer duration of effect with a decreased risk of infection [20]. While there exist technical descriptions of the cryoneurolysis of various nerves, the present report, to our knowledge, is the first to describe the use of ultrasound and fluoroscopic guided percutaneous cryoneurolysis of the subcostal nerve [15]. Although many treatment options are available for the management of chronic thoracic and abdominal pain, the present case demonstrates promise for cryoneurolysis of the subcostal nerve to become a potential fixture in the pain management pathway.

## Conclusions

Cryoneurolysis of the subcostal nerve should be considered after conventional methods have demonstrated limited efficacy. A thorough workup should be performed to assess whether a patient’s pain is indeed the result of subcostal nerve pathology. Further research is warranted to explore the long-term effects of cryoneurolysis, as well as its heterogeneity or lack thereof, in the treatment of peripheral mononeuropathies.
